# Measurement of Human Flourishing Constructs in Evaluation of Voluntary Family Planning Interventions: A Modified Scoping Review of the Literature

**DOI:** 10.3390/ijerph23080998

**Published:** 2026-07-30

**Authors:** Ahna Ballonoff Suleiman, Claire Cole, Selena Regalado

**Affiliations:** 1 Center for Regional Change, University of California Davis, Davis, CA 95616, USA; 2 Transformative Story, Totnes TQ9 6BW, UK; claire@transformativestory.net

**Keywords:** voluntary family planning, human flourishing, program evaluation, contraception, modified scoping review

## Abstract

**Highlights:**

**Public health relevance—How does this work relate to a public health issue?**

- Voluntary family planning (VFP) serves as a tool for people to prevent unintended pregnancy, and control healthy timing and spacing of pregnancies.
- The conditions to thrive, including confidence, supportive relationships, and resources, are a focus of human flourishing research and are increasingly recognized as important to public health outcomes.

**Public health significance—Why is this work of significance to public health?**

- Healthy timing and spacing of pregnancy, supported by VFP, is associated with improved maternal and newborn health outcomes.
- VFP interventions have evaluated performance in advancing relevant conditions for thriving without leveraging the scholarship of human flourishing.

**Public health implications—What are the key implications or messages for practitioners, policy makers and/or researchers in public health?**

- Reducing duplication and maximizing integration between the fields of VFP and human flourishing matters acutely in the current moment, as cuts in global health and development aid funding require the field to do more with less.
- Shifting the design and evaluation of VFP interventions to include components of human flourishing can improve lifelong health and wellbeing in tandem with sexual and reproductive health.

**Abstract:**

Managing one’s fertility and determining the timing of one’s pregnancies is integral to human flourishing. An emerging body of literature focuses on global measurement of human flourishing. Despite significant debates in the field, the goal of measuring human flourishing is to understand what it means to live life well. We conducted a modified scoping review of quantitative, qualitative, and mixed-method grey and white literature to identify the extent to which evaluations of interventions aiming to increase the use of voluntary family planning include measures of human flourishing. We searched for peer-reviewed articles published from 2007 to 2025 across four databases (PubMed, Google Scholar, Web of Science, and Science Direct). We completed a full-text review of 583 documents and identified 111 that contain both a behavioral measure of contraceptive use and the measurement of at least one flourishing construct. We captured both the flourishing constructs and measures used. The most measured flourishing constructs were self-efficacy, shared decision-making, social support, mental health, and agency. Less frequently, evaluations measured happiness/pleasure/joy, empowerment, emotional intimacy, physical health, meaning/purpose, social capital, autonomy, trust, and wellbeing. Among the 111 articles, 76 (68.5%) of them included both flourishing-related intervention components and evaluation measures. Twenty-three measured flourishing-related outcomes in the evaluation but did not explicitly include flourishing components in the intervention. Collectively, these findings suggest that there is room for growth to fully understand the relationship between voluntary family planning interventions and the construct of human flourishing.

## 1. Introduction

Childbearing and reproduction are among the most core functions of human life. Planned, desired childbearing can support parents, children, families, and societies to thrive. Research on human flourishing has demonstrated that reproduction and childbearing are core inflection points through which bonding forms, character develops, and meaning and purpose is cultivated—with lifelong effects [1,2]. Childbearing also marks the transitions of multiple generations as they evolve in their familial and social roles. As individuals become parents, parents become grandparents, and members of each generation shift in their roles to protect and nurture the next generation [3].

Conversely, childbearing that occurs under adverse conditions has similarly profound, negative implications for flourishing. Unplanned and unwanted pregnancies are equally as common as planned, desired ones. Unintended pregnancies comprise 50% of pregnancies in the world today, many of which are unwanted [4]. Frequent, mistimed, and early pregnancy and childbirth are linked to a multitude of negative health outcomes, including various maternal health morbidities [5,6,7]. Complications during pregnancy and delivery are a leading cause of mortality among adolescent girls, and children of young mothers face elevated rates of poor nutrition, adverse health and wellbeing, and mortality [8].

Since the dawn of the field of philosophy, there has been debate about what it means to live a good life or flourish. The field of human flourishing often points to Aristotle’s definition of eudaemonia—wellbeing, fulfillment, and satisfaction—as well as hedonia—the experience of pleasure or happiness—as the core components of human flourishing. Over the past 10 years, the field of positive psychology has seen numerous, often conflicting, attempts to theoretically define human flourishing, and scholars have gone to great lengths to distinguish flourishing from the broader construct of wellbeing [2,9]. Recently there has been a concerted effort in the field of flourishing to develop a more rigorous theoretical approach, including conceptual frameworks and measures such as those in the Global Flourishing Study [10,11,12]. While there is no universally applicable meaning for human flourishing, based on our review of the literature to date, for the purposes of this review, we have adapted and use Willen et al.’s 2021 definition of human flourishing, developed for the context of global health and migration, as “an active process of striving to live in keeping with one’s defining values, commitments and vision for the future, as individuals and in the context of one’s family and the communities to which one belongs” [13]. Flourishing is informed by cultural expectations and social relationships, and influenced by the social, political, and economic structures that shape people’s lives.

Given the central role of sexual health and sexual behavior in determining the timing of conception, childbearing, and parenting, access to contraception through voluntary family planning (VFP) programs is a central tenant of global sexual reproductive health and rights (SRHR). The evidence is clear that people use contraception as a tool to improve their health and wellbeing, and their ability to flourish in life [14]. Conversely, many health behavior theories used to inform the design of VFP interventions include constructs related to flourishing (e.g., social support, agency, empowerment) as essential parts of the pathway to contraceptive adoption [15]. Reflecting this bidirectional relationship, there has been a move towards more holistic frameworks for sexual and reproductive health (SRH) to look beyond counting the number of new contraceptive users and to articulate the value of wellbeing in public health and develop metrics that can be used to quantify and measure wellbeing-related constructs and outcomes [16,17].

There is significant overlap in the conceptual frameworks of human flourishing and SRHR. The Flourishing and Secure Flourish indices include many domains that are essential for SRHR, including close social relationships, meaning and purpose, financial and material stability, physical and mental health, happiness and life satisfaction, and character and virtue [2,13]. Similarly, VFP and SRHR frameworks include constructs including belonging, autonomy, resilience, character, and positivity, which impact the ability to plan and achieve one’s reproductive goals [16,17]. Despite the resonance of these theoretical frameworks and their robust empirical evidence, there is limited integration between the evidence bases of human flourishing and VFP. Historically, VFP researchers and practitioners have struggled to advocate for and implement comprehensive interventions that meaningfully address and bolster VFP, SRHR, and human flourishing. Resource constraints and prioritization of lean, high-impact interventions have led VFP interventions to primarily prioritize client outreach and direct contraceptive service delivery, aiming to enhance the knowledge of and access to contraceptives. By contrast, investments targeting the underlying and upstream drivers of reproductive decision-making and related health and flourishing outcomes have largely become underemphasized [18].

However, global data makes its case for reconceptualizing the focus of VFP and SRHR investments and programming. At the time of this review, lack of awareness of and access to contraceptive commodities were no longer the primary barriers to individuals’ ability to plan the families they desire globally. Rather, stigma and active opposition to the agency, self-determination, and autonomy of people who can become pregnant were among the leading barriers to contraceptive use. Importantly, these barriers surpassed historical challenges of awareness of or access to contraceptive services [4]. Since we began this review, a suite of recent US policy changes profoundly changed the global SRH funding landscape and created a public health emergency of international concern, threatening the autonomy, health, and wellbeing of birthing people and creating new threats to contraceptive access and SRH world-wide [19,20]. While reinstituting access to information and contraceptive commodities is essential, this also suggests that the primary requirement for VFP strategies is no longer solely the advancement of education about and access to contraceptives, but also it is also critical to strengthen support structures to create conducive environments for individuals and couples to shape and achieve their reproductive goals, including planning their desired families. This requires investments in domains of human flourishing such as empathy, belonging and social relationships, character, agency, and autonomy.

Integrating concepts of wellbeing and thriving into SRH interventions is not a new concept. For many years, researchers, funders, and program staff have understood that people use contraception as a tool to improve their health and wellbeing and many health behavior theories also include flourishing-related components (e.g., social support, agency, empowerment) as essential parts of the pathway to adopting health-protective behavior. In addition, there is also clear evidence that managing the timing and spacing of childbearing through contraceptive use supports positive health, social, and economic outcomes, which supports wellbeing [21]. While the newer Sexual and Reproductive Wellbeing (SRWB) conceptual model and measurement scale has done extensive work to move the field of contraception beyond measuring the number of people using contraceptive methods towards more person-centered measurement of SRWB, they have done this without integration with the body of evidence from the human flourishing literature.

To understand the conceptual overlap and span the silos between VFP interventions and human flourishing constructs in the design and evaluation of existing VFP interventions, we conducted a modified scoping review of the literature [22]. We began with the intention of conducting a systematic review of the literature but encountered two key challenges. First, there is broad diversity of conceptual definitions of human flourishing and lack of consensus about how the construct can be most effectively measured. Second, there is significant diversity in the way that flourishing-related constructs are measured in VFP program evaluations and many include both quantitative and qualitative measures. In contrast to a systematic review which attempts to evaluate the quality and risk of bias in data in an existing area of inquiry, the goal of a scoping review is to map the existing evidence on a topic and identify the main concepts and constructs that appear in the literature [22]. In line with this methodology, we conducted a modified scoping review to understand the extent and range of measurement of human flourishing constructs in VFP program evaluations and understand the diversity of constructs and measurement tools [22]. This allowed us to integrate insights from a heterogenous field of mixed-method evidence that did not lend itself to a systematic review. We conducted this modified scoping review to bridge the silos between the fields of VFP and human flourishing to optimize the use of limited resources to improve human health.

## 2. Methods

From December 2024 through June 2025, we conducted a modified scoping review of the literature. We began by conducting a narrative review of literature and frameworks on VFP and human flourishing. From this narrative review, we identified a set of overlapping constructs (see Table 1) that appear in theoretical frameworks for both VFP and human flourishing. 

We reviewed the white and grey literature of quantitative, qualitative, and mixed-method English language program evaluations of VFP and contraception interventions to identify those that included a behavioral measure of contraceptive utilization and captured one or more human flourishing construct. We focused our review on evaluations of interventions that aimed to increase the use of VFP, including HIV prevention interventions that included condom use if the condom use could offer dual protection for pregnancy prevention. We searched for peer-reviewed articles published from 2007 to 2025 across four databases (PubMed, Google Scholar, Web of Science, and Science Direct) for articles that included at least one MeSH search term related to program evaluation (e.g., “Family Planning Program Evaluation”, “Program Evaluation”, “Program Effectiveness”) and one MeSH search term related to contraception (e.g., “Contracepti*”, “Contraceptive agents”, “Contraceptive methods”, “Contraceptive Usage”), family planning, condom, natural family planning, and lactational amenorrhea. The complete list of search terms is available upon request. We simultaneously reviewed evaluations of VFP programs captured in grey literature that did not appear in peer-reviewed journals. Based on our expertise in the field, recommendations from experts in the field, and additional Google searching, we developed a list of 59 organizations, agencies, and collaboratives that implement VFP interventions across the globe. We conducted a thorough search of each organization’s website and downloaded VFP program evaluation reports, fact sheets, and other program evaluation documentation to include in our review. We uploaded all matching articles into Coevidence and used it to manage the review of the documents.

ABS led the extraction of the white literature and CC and ABS conducted the review of the grey literature. ABS and SR uploaded all eligible articles into Coevidence. The search for grey and white literature yielded 2009 documents (excluding duplicates).

We reviewed the title and abstract to confirm that each document met the criteria of the evaluation of a contraceptive intervention and excluded 1425 documents. CC, ABS, and SR completed the title and abstract screening and full-text review. We began working as a full team to screen titles and abstracts. Once we had refined our screening criteria and reached a consensus on which articles should be included and excluded, we reduced our efforts to one screener per article. If we could clearly determine if the manuscript should be included or excluded, the screener did so in Coevidence. If we could not make a clear determination, we flagged the article and collectively reviewed and screened it during weekly meetings throughout the review process.

We then reviewed the full text of the remaining 584 documents to determine if they contained both: (1) a behavioral measure of contraceptive use, and (2) the measurement of at least one construct related to human flourishing or wellbeing, including happiness, flourishing, empowerment, emotional intimacy, shared decision making, mental health, physical health, meaning or purpose, self-efficacy, feeling valued, social support, social capital, autonomy, agency, trust, wellbeing, pleasure, and joy. We followed similar procedures for full-text review and began reviewing articles in meetings with all members of the team. Once we had reached a consensus on inclusion criteria and consistency in evaluation, we reduced to having two members of the team independently read and review the articles and tracked their inclusion decision. If the reviewers agreed, their inclusion or exclusion designation was followed. If the reviewers disagreed, we discussed those articles during weekly meetings.

**Figure 1:**
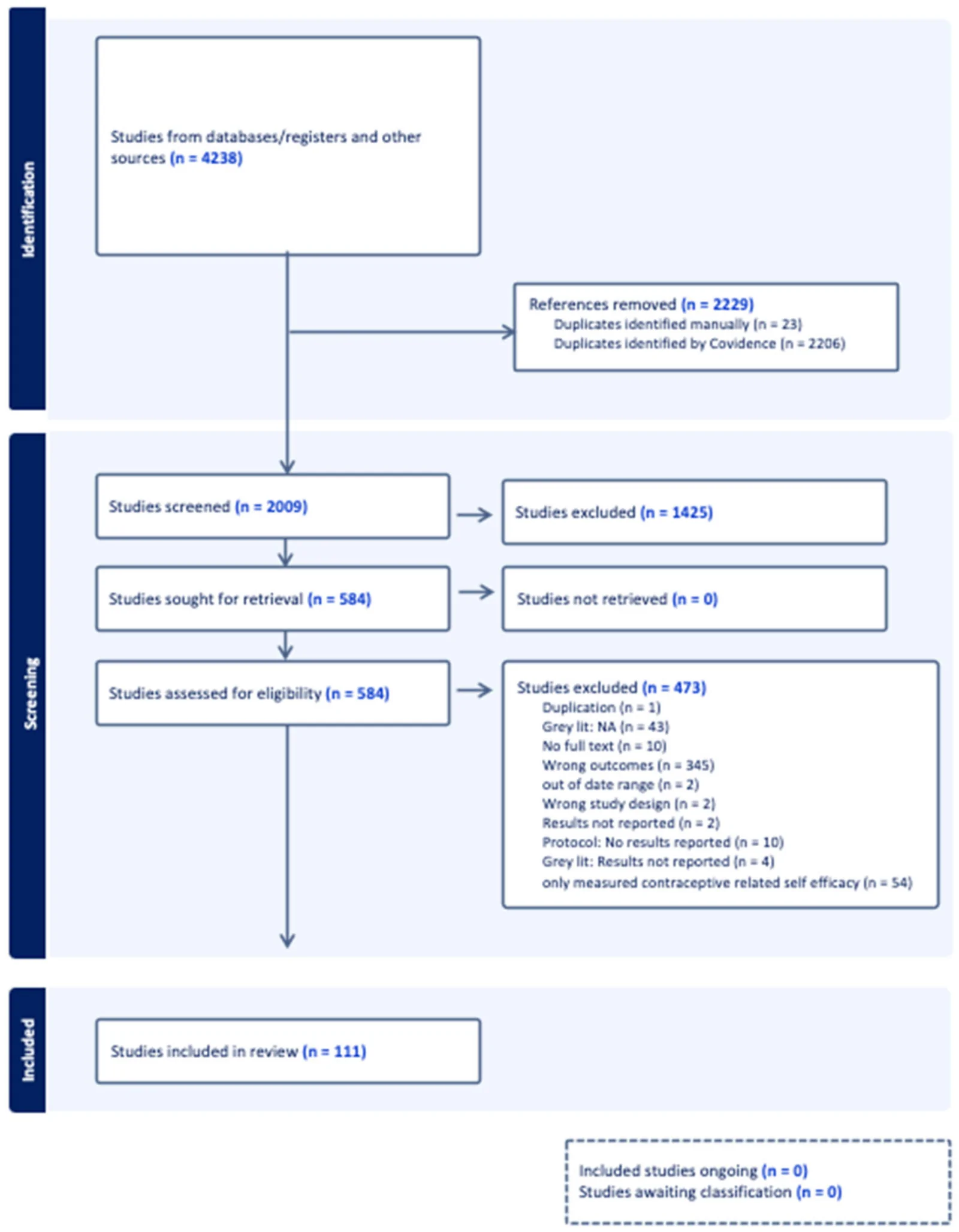
PRISMA flowchart for the literature review.

From the full-text review, we identified 165 evaluations of contraception interventions that included measurement of at least one human flourishing construct. The majority of them (*n* = 122; 73.9%) measured self-efficacy, and in 58 of those (47.5%), self-efficacy was the only measured human flourishing metric. This prompted us to re-review articles we had included with self-efficacy as the only human flourishing outcome and exclude articles that included only measurement of condom use or contraceptive use self-efficacy or self-efficacy related to negotiating contraception or condom use with a partner, as we determined that, alone, these were too domain-specific and not sufficient measures of human flourishing constructs. This led us to exclude an additional 54 manuscripts. Upon completion of this review, 111 manuscripts remained. The full scope of the literature review is captured in the PRISMA flowchart (Figure 1) below and the PRISMA-ScR Checklist is available in the Supplementary Materials for this article (see end of manuscript for link) [22].

Two people reviewed each of the final 111 manuscripts and extracted and documented which human flourishing constructs were measured and what scales were used in the measurement. Our goal was not to assess the quality of the measurement, but rather the activity of measurement. During the full-text review, we used Excel (version 16.111.1) to document: (1) the context of the intervention; (2) whether the intervention explicitly included HF components in the intervention design; (3) the contraceptive measures and outcomes; and (4) the wellbeing indicators, measures and outcomes. Each reviewer had a single worksheet in the Excel file where they documented their extracted data. SR then integrated the data from the two reviewers for each manuscript in a single worksheet, indicating when reviewers differed in their findings. If reviewers differed in their extraction and documentation, the article was reviewed and discussed by the full team until we reached a unanimous decision. The full protocol for article selection and data extraction is available upon request.

## 3. Results

The contraceptive evaluations clearly prioritize many constructs related to flourishing and wellbeing in intervention design and evaluation. At the same time, defining, operationalizing, and measuring these constructs proved to be challenging. Among the 584 full-text manuscripts that we reviewed, one quarter of them (*n* = 145) described VFP interventions that explicitly included constructs related to human flourishing. Just over half of these (*n* = 76) articles included human flourishing components in both the intervention design and evaluation. The remaining half of the interventions that include human flourishing components in their design did not try to measure related outcomes. In fact, in our review, we found that programs that talked the most about including human flourishing components in their program design often only measured behavioral, method-adoption-related outcomes such as the number of contraceptive users, which were tangential and insufficient to understand the interventions’ intended contributions to flourishing.

We identified 111 evaluations of contraceptive interventions that measure at least one human flourishing construct (see Table 2). The most common human flourishing constructs we observed, in order of prevalence, are self-efficacy, shared decision-making, social support, mental health, and agency. Less frequently, the literature also included reports of constructs related to happiness/pleasure/joy, empowerment, emotional intimacy, physical health, meaning/purpose, social capital, autonomy, trust, and wellbeing.

Table 3 captures the frequency of measurement of human flourishing-related constructs we found in the VFP literature. Among the 111 final articles, 23 measured human flourishing-related outcomes in the evaluation but did not explicitly include human flourishing components in the intervention. Seventy-six (67.9%) of them included both human flourishing-related measures and intervention components. In the remaining twelve articles, we were unable to determine if the intervention contained human flourishing components based on the available intervention description. Below, we discuss the primary constructs that appear in the literature.

### 3.1. Self-Efficacy

It is well established that self-efficacy is a core component of contraceptive interventions [133,134]. Reflecting this, over half (*n* = 59; 53.1%) of the articles we reviewed measured self-efficacy. Most of the measurement of self-efficacy in the contraceptive literature is domain-specific, related to contraceptive method self-efficacy or self-efficacy in negotiating condom or contraceptive use with a partner. While we excluded articles that included these types of self-efficacy as a sole measure of human flourishing, if it was measured in addition to other human flourishing metrics, we included it in this review. Of the 59 articles that included a measurement of self-efficacy, 29 (49.2%) focused on contraceptive use self-efficacy. The remaining articles included measurement of self-efficacy to prevent unwanted actions (e.g., self-efficacy related to unwanted sexual attention, self-efficacy to avoid unwanted or unprotected sex); self-efficacy to access family planning services; self-efficacy to make sexual decisions; or self-efficacy to communicate with partners about sex or contraception. Only eight of the articles reported measurements focused on generalized self-efficacy or measured individuals’ self-efficacy to pursue desired aspects of their SRH lives (e.g., self-efficacy in wanted and safe sex; confidence in the ability to give sexual consent), aspects which might align with a flourishing life.

### 3.2. Social Support and Shared Decision-Making

Two other predominant human flourishing metrics measured in the VFP literature are social support and shared decision-making. Social support appears in the literature at the individual level as support from peers, partners, family members, and health care providers, and at the community level as social cohesion, collective efficacy, social inclusion, community acceptance and general community support for people who can become pregnant, including cisgender girls and women, transgender men, and non-binary individuals. Shared decision-making appears in the literature primarily as communication with sexual partners and/or joint decision-making. It less frequently appears as communication with parents (for adolescents) or peers. Additional constructs related to social relationships that we observed in the evaluations of VFP programs were emotional intimacy, relational meaning or purpose, feeling valued, social capital, trust, and pleasure. In the articles we reviewed, less than 10% included a measure of sexual pleasure and only 1% measured happiness.

Many of the interventions we reviewed in the literature focus on improving relationships between people and their health care providers, health educators, peer educators, teachers, or other providers of information and services. At times, the goal is to reduce stigma among providers to improve client/patient satisfaction or experience, particularly towards adolescents, low-income, or other marginalized populations, which has been shown to have some positive effects on contraceptive adoption and service utilization. Some of the process and qualitative evaluations explore how the quality of relationships between providers and clients/patients leads to improved feelings of social support, comfort with accessing contraceptive information and services, and ultimately contraceptive uptake and use.

For many people, particularly those who live in cultural settings where extended family influences decisions around SRH and VFP, parental relationships can significantly impact contraceptive use and experiences of flourishing—particularly for adolescents and young adults. In the literature review, evaluations measured parental support using metrics related to communication with parents, living with parents, parent–child relationship quality, and perceived social support from parents. Aligned with this, many of the interventions included components aiming to improve the quality of parent–child relationships; communication between parents and children about sexual behavior and contraception; and parents’ knowledge, attitudes, and beliefs around contraceptive use. In the interviews, experts largely discussed the importance of positive relationships with parents in the context of adolescent and youth sexual health and wellbeing. For youth with non-traditional families (i.e., orphans, foster youth) the parental relationship can extend to a relationship with a non-biologically related, trusted adult.

### 3.3. Mental Health and Empowerment

In our review of the human flourishing literature, we found relevant insights that one’s relationship with and sense of self are foundational for flourishing, including building positive relationships with others. This literature suggests that one’s mindset and perspective informs how one perceives and experiences their relationships and, in turn, their own flourishing [9]. The theoretical frameworks underlying SRH interventions indicate that one’s experience of self-determination informs contraceptive use and impacts one’s ability to form positive relationships, including romantic and sexual relationships, with other people [135,136,137]. Twenty-three of the manuscripts (20.7%) included some measure of mental health and 15 (13.5%) included a specific measure of empowerment. Measurement of mental health included sense of wellbeing, happiness, inversely as lack of sadness or depression, being positive about the future, and life satisfaction. Measures of empowerment included generalized empowerment, economic empowerment and empowerment in romantic and sexual partnerships.

### 3.4. Agency and Autonomy

Some measurement of agency and/or autonomy appeared in 20 (18.0%) of the articles. We used a broad definition of agency as we evaluated this construct in the literature. Among the top four human flourishing constructs in the literature, measurements of agency were the least cohesive and least prevalent. The most consistent metric associated with agency was reproductive decision-making agency. We classified additional metrics under agency including power over versus power with, civic action, confidence, assertiveness, perceived control, decision-making power, power in relationships, and mobility. While we recognize the imprecision of putting these distinct concepts into one category, they centered on a common idea about people having some sense of independence and control. Autonomy was sometimes described as distinct from agency and sometimes included in measures and scales of agency. At the same time, the importance of distinguishing between agency and autonomy was often addressed, particularly in non-white, non-Western, lower-resource settings. While autonomy places the highest value on the individual, the concept of agency includes and elevates more relational or collective wellbeing, which is important both for SRH and human flourishing.

## 4. Discussion

Many human flourishing constructs exist in the SRH theory and evidence base—both informing the design of interventions and the evaluation of program outcomes. Alongside measuring contraceptive uptake, self-efficacy, shared decision-making, social support, mental health and agency all appear in VFP program evaluations, suggesting that they are seen as essential parts of the pathway to effective contraceptive use. At the same time, the fact that 47.6% of interventions that described flourishing constructs as driving their theory of change or program design did not then include them in their evaluation measurement suggests that there is still work to be done to improve measures and measurement of flourishing-related outcomes in VFP interventions—even among interventions already oriented toward these constructs by design.

The overwhelming majority (*n* = 473; 81.0%) of evaluations of contraceptive interventions we reviewed did not measure any construct related to flourishing, limiting their contribution to understanding the value that VFP interventions have added to the overall sense of flourishing in people’s health and lives. Considering that our inclusion criteria required the presence of a human flourishing-related outcome, this proportion underscores that our analysis focuses on a subset of the VFP field that is engaged with flourishing and wellbeing, distinct from the larger field of VFP and its evaluation practices.

Of those evaluations that did include a flourishing measure, the constructs that were least often measured are important to note. Happiness, pleasure, joy, empowerment, emotional intimacy, meaning, purpose, social capital, and trust are key dimensions of healthful and flourishing life. Given the capacity for childbearing and parenting to serve as both a source of flourishing as well as hardship, in part depending on timing and spacing, it is important to consider the relationship between contraceptive use and flourishing for people who can become pregnant.

As young people navigate adolescence, one of their primary tasks is to navigate the social, emotional, and biological transitions that occur with the onset of reproductive capacity. Data from around the globe suggests that adolescence serves as a key inflection point that can lead to positive or negative lifelong trajectories [138]. If young people learn to use contraception and manage their reproductive capacity in a way that promotes flourishing, it has the potential for positive impact throughout the life course. For this learning to support positive developmental trajectories, there is clear need for interventions that support both desired contraceptive use and inner capacities for a positive developing sense of meaning, purpose, and place in the world, including experiences of pleasure, joy, emotional connection, and intimacy. As our review finds, many interventions have sought to pursue some aspects of this combined focus over the last 18 years. But the majority have not assessed their performance against these aims.

Overall, our review demonstrates insufficient evidence to understand VFP interventions’ contributions to constructs of human flourishing despite frameworks and guidance underscoring their importance to positive outcomes for people who can become pregnant and their families. The reasons for this are multi-fold. As noted above, there may be insufficient commitment to assess these contributions, making it difficult to advance accountability toward these dimensions of health and wellbeing. In addition, limitations to the feasibility of evaluating such constructs also contribute to the evidence gap.

As we look for ways to integrate the theoretical frameworks of human flourishing and SRH, we recognize the need for standard measures that capture the breadth of human flourishing constructs that are relevant to SRHR. Longstanding scholarship on the measurement of relevant constructs like agency, autonomy, and empowerment cautions against treating such concepts as static or universal, emphasizing the importance of sensitivity to context including gender, race, and class in the social and structural environment, as well as the centrality of the subjective experience in assessing wellbeing [139,140,141]. Any effort to offer standardized measures must, then, be done in ways that are responsive to these dimensions rather than assuming global relevance. No single-measurement approach can adequately reflect flourishing and wellbeing inclusive of SRHR for all young people, across all contexts. It will be important to identify the pathways and the mechanisms that link these two fields, as well as continue efforts to draw attention to the importance of flourishing and wellbeing-related dimensions of life to overall health and development agendas.

Despite efforts in the last two years to significantly thwart equitable health and wellbeing for all people who can become pregnant, this work is still happening. Three examples are Dehlendorff and colleagues’ work to advance the SRWB framework [16,142]; Kumar and colleagues’ work to recenter positive birth experiences in systems strengthening [143]; and the Pleasure Project’s work to advance pleasure as part of sexual health, rights, and wellbeing [144,145]. In the case of the SRWB movement, their efforts to ensure precision and effective measurement have focused on integrating wellbeing measures into evaluating the quality of clinical SRH health care [16]. Their efforts have made a great impact on the SRH field as they have convinced donors and other partners about the importance and feasibility of a wellbeing lens in clinical SRH care.

The findings from across our review of the VFP literature and VFP programs point to the potential opportunity to enhance both the fields of human flourishing and SRH through better integration to improve the lives of people who can become pregnant. At the same time, there is a fundamental tension in how this can be done. The field of SRH focuses on intervention and praxis, whereas the field of human flourishing prioritizes measurement and observation. This is a common tension between public health and other social sciences (e.g., sociology, anthropology), and begs the question of whether measurement and observation are sufficient or, when the purpose of a field’s work is to enable lives to improve, if there might not be a shared moral responsibility to orient towards addressing inequities in outcomes such as flourishing, inclusive but beyond clinical health outcomes.

## 5. Conclusions

Our review joins other recent work highlighting the importance, opportunities, and challenges of integrating constructs relevant across SRH and flourishing. People who can become pregnant deserve support for their ability to choose whether and when to become parents as a foundation for a flourishing life. Yet, as this review demonstrates, in the last 18 years, VFP interventions have largely failed to assess, and therefore to be accountable for, their contributions to these dimensions of life. It is important to note that a limitation of this research is that we conducted this search in Spring 2025, during a time when the United States government instituted a funding freeze and review of all grants and programs related to gender and women’s health, prompting many organizations to remove online documentation, including of VFP programs, from the public domain [146,147]. We used tools to make the search as thorough as possible, and we recognize this likely limited our findings. A second limitation of this research is that it is possible that programs collected data on human flourishing-related constructs that was not reported due to a lack of positive findings and/or a lack of space allotted in peer-reviewed manuscripts. As such, it is possible that some of these evaluations collected data on flourishing constructs that did not appear in the literature. A third limitation, as discussed above, is that our inclusion criteria intentionally narrowed our analysis to focus on the subset of the VFP field already engaged with flourishing and wellbeing rather than on VFP evaluation practice broadly. A randomly sampled or representative review could offer a field-wide perspective. We also note that, consistent with the scoping review methodology, we did not assess the quality or risk of bias of the included studies. Future analyses may benefit from incorporating this, as well as conducting interpretative analysis to observe patterns in the flourishing and VFP literature considering region, population subgroups, and sociopolitical contexts and explore why certain flourishing-related constructs remain underrepresented. Finally, the measures and methods used to collect data across the literature were highly variable. While the inclusion of quantitative and qualitative data supported our goal of understanding the breadth of the literature, it also created challenges in synthesizing the literature. This was particularly true for qualitative reports, where we were limited by the themes the authors presented and may have missed reports of flourishing-related findings.

Reducing duplication and maximizing integration between the fields of VFP and human flourishing matters acutely in the current moment, as recent seismic shifts in global health and development aid funding are requiring the field to do more with less. It is ever more critical that VFP interventions are designed to deliver holistic value for the people they serve. This is especially true for young people who can become pregnant, who represent the world’s future. Adolescence is a critical period of social and emotional expansion, and the inner capacities young people develop during this time—for joy, intimacy, meaning, and a developing sense of purpose and place in the world—are inseparable from their sexual and reproductive health. Realizing this potential requires a broader view of flourishing in VFP programming that extends beyond clinical outcomes to encompass the fuller dimensions of a life well-lived. This demands a shift in how interventions are designed, measured, and evaluated: toward approaches that are participatory, attentive to lived experience, and oriented by a shared commitment to young people’s flourishing, inclusive but beyond their health.

## Figures and Tables

**Table 1:** Overlapping constructs in theoretical frameworks of human flourishing and VFP.

Individual Level	Interpersonal Level
Self-esteem Self-confidence Mental health Psychological wellbeing Agency Self-efficacy Purpose Empowerment	Supportive relationships Connection/Contribution Collective efficacy/engagement with life Social capital

**Table 2:** Articles and reports about evaluations of contraceptive interventions with measurement of one or more human flourishing constructs.

Article [Reference]	Study Design	Country	Research Language(s)	Key Outcomes	Sample Size
Abe, et al., 2016 [23]	Cluster RCT	USA	English	SRH knowledge, behavior, attitudes, skills, intentions	2203
Accelerating Universal Access To Family Planning Celebrating the Legacy of the Shukhi Jibon Project in Bangladesh, 2024 [24]	Analysis of population level data pre–post intervention	Bangladesh	Not specified	Family planning service delivery, contraceptive use	N/A
Adeyeba, et al., 2023 [25]	Randomized parallel group	USA	English	Number of sexual partners, condom use, STIs, self-efficacy	203
Advancing Sexual and Reproductive Health and Rights Among Rohingya and Host Communities in Cox’s Bazar, Bangladesh: Lessons from a Project for Continued SRHR Support, 2023 [26]	Analysis of population level data pre–post intervention	Bangladesh	N/A	Family planning service delivery, contraceptive use	N/A
Ajayi, et al., 2021 [27]	Mixed-method treatment-only group	Kenya	Swahili, English, Dholuo, Maasia	Use of SRH health services, contraceptive use	1110-quant 45-qual
Anastario, et al., 2013 [28]	Mixed-method treatment-only group	Barbados	English	Condom use behavior, attitudes, and norms, Alcohol use	256-quant 15-qual
Aparicio, et al., 2019 [29]	Mixed-method quasi- experimental	USA	English	Linkage to SHR care, contraceptive use	51
Aronson, et al., 2013 [30]	Treatment-only group	USA	English	Condom use, attitudes, knowledge, beliefs and self-efficacy	57
Asheer and Kisker, 2014 [31]	Mixed-method observational	USA	English	Contraceptive use, program effectiveness	160
Babalola, et al., 2019 [32]	Cluster RCT	Nigeria	Hausa	Desired family size, communication self-efficacy, spousal communication, misinformation rejection, contraceptive use	565
Barnet, et al., 2007 [33]	RCT	USA	English	Parenting attitudes, condom access, contraceptive use, depression	84
Brawner, et al., 2021 [34]	RCT	USA	English	HIV/STI knowledge, condom use, number of sexual partners	108
Brown, et al., 2011 [35]	Cluster RCT	USA	English	Sexual behavior, knowledge and attitudes	185
Calsyn, et al., 2013 [36]	Mixed-method delayed-treatment group comparison	USA	English	Condom use	202-quant 66-qual
Can Light-touch Enhancements Improve Postpartum Family Planning Use Among First-time Mothers: Findings from small-scale testing of an integrated approach in Tanzania, 2022 [37]	Pre–post No control	Tanzania	Not specified	Contraceptive use	351
Center on Gender Equity and Health, 2023 [38]	Cluster RCT	Burkina Faso Niger Mali	Not specified	SRH and gender norm attitudes, behavior and knowledge, contraceptive use, adolescent birth rate	2250
Center on Gender Equity and Health, 2022 [39]	Cross-sectional cluster RCT	Burkina Faso Niger Mali	Not specified	SRH and gender norm attitudes, behavior and knowledge, contraceptive use, adolescent birth rate	2244
Chinman, et al., 2018 [40]	Cluster RCT	USA	English	Condom and sex knowledge, attitudes, and behavior	477
Chowdhary, et al., 2022 [41]	Qualitative, cluster, focus groups and interviews Treatment group only	Ethiopia	Amharic	SRH and contraception knowledge and behaviors	320
Dalaba, et al., 2016 [42]	Qualitative, cluster, focus groups and interviews Treatment group only	Ghana	Kasem Nankam	SRH and contraception knowledge and behaviors	24
DiClemente, et al., 2014 [43]	RCT	USA	English	SRH and contraception knowledge, behaviors, and self-efficacy, STI infection	188
Doyle, et al., 2018 [44]	RCT	Rwanda	Kinyarwanda	Interpersonal violence, male involvement, contraceptive use, gender equity	1199
Dunbar, et al., 2014 [45]	RCT	Zimbabwe	Not specified	Economic and social empowerment, SRH behavior, gender based violence, relationship power	315
Edmeades, et al., 2014 [46]	4-group RCT	Ethiopia	Not specified	Economic behavior, gender-based violence, partner communication, SRH knowledge & behavior, social capital & support, use of SRH services & contraception	Not specified
Efficacy of CBT to Support Postpartum Mental Health and FP in Ethiopia, 2024 [47]	Cluster RCT	Ethiopia	English Amharic	Depression and anxiety, contraception use, behavior and enablers related to family planning	Not specified
End of Project Evaluation: Peak youth tackling HIV project: Restless Development Zimbabwe, 2020 [47]	Mixed-method	Zimbabwe	Not specified	SRH knowledge, use of SRH services, confidence, knowledge & skills	255-quant 60-qual
Erulkar and Muthengi, 2009 [48]	Quasi-experimental pre–post	Ethiopia	Amharic	Social and educational participation, marriage age, SRH knowledge, contraceptive use	926
Essien, et al., 2011 [49]	RCT	Nigeria	English	HIV/AIDS knowledge, contraceptive use	346
FACT: Fertility Awareness for Community Transformation: EDEAN “Let’s Come Together and Strengthen Child Spacing”: Pilot Snapshot, 2020 [50]	Mixed-method quasi- experimental pre–post	Uganda	English Acholi	Fertility awareness, contraceptive access and use	600-quant Qual not specified
FACT: Fertility Awareness for Community Transformation: WALAN: Wake ki Lago Nywal, 2016 [51]	Qualitative interviews post implementation	Uganda	English Acholi	Fertility awareness, contraceptive access and use	16
Feldman, et al., 2009 [52]	Cluster RCT	Mexico	Spanish	Contraceptive use, birth spacing, autonomy	8568
Foss, et al., 2022 [53]	RCT	USA	English	Sexual and contraceptive behavior, comfort with sexuality	810
Givaudan, et al., 2007 [54]	Quasi-experimental pre–post	Mexico	Spanish	Condom knowledge, use, and self-efficacy, communication, HIV knowledge	2064
Gómez-Lugo, et al., 2022 [55]	Cluster RCT	Colombia	Spanish	Age of sexual debut, number of sexual partners, condom use, HIV attitudes, knowledge and self-efficacy	2047
Gong, et al., 2009 [56]	Cluster RCT	Bahamas	English	Condom use knowledge, attitudes, and behavior	1360
Harper, et al., 2009 [57]	Quasi-experimental	USA	English	Condom use, abstinence, number of sexual partners, HIV/AIDS and STI knowledge, self-esteem, communication,	378
Hattori, et al., 2010 [58]	Cross-sectional post exposure no control	Lesotho Mozambique Uganda Zambia	Sesotho English Portuguese Luganda Runyanole-Rukiga Runyoro- Rutoro Nyanja Bemba	Trust of sexual partners, condom use	1738
Hémono, et al., 2024 [59]	Mixed-method treatment group only	Rwanda	Kinyarwanda	Contraceptive use, partner communication, sexual behavior, SRH knowledge, gender based violence	158-quant 28-qual
Herrling and Hirsch 2022 [60]	RCT	USA	English	Condom and contraceptive use, communication about contraception	223
Jåmiä et al., 2024 [61]	Scoping Review	Multiple	Multiple	Advancement of SH abilities, sexual behavior, sexual identity	N/A
Jennings, et al., 2014 [62]	Quasi- experimental nonrandomized matched comparison group	USA	English	Sexual risk reduction skills, communication about sex and contraception, sexual boundaries, sexual activity, contraceptive use	157
Jihyun and NamCho, 2017 [63]	RCT	South Korea	Korean	Contraceptive knowledge, self-efficacy, control, communication, autonomy, contraceptive use	48
Kachingwe, et al., 2019 [64]	Qualitative post-exposure	USA	English	Contraceptive use, satisfaction with clinical services, knowledge	11
Kalmuss, et al., 2008 [65]	Non-equivalent control group	USA	English	Use of health care services, SRH knowledge and attitudes, number of sexual partners, condom use	231
Kaponda, et al., 2011 [66]	Cluster quasi- experimental random sampling	Malawi	Chichewa	Condom attitudes, self-efficacy, partner communication, condom use, community prevention activities	2242
Kedde, et al., 2018 [67]	Mixed-method pre–post treatment group only	South Africa	English	Gender equitable attitudes, attitudes towards contraceptives, condom use	265-quant 33-qual
Kedzior, et al., 2020 [68]	Systematic Review	Multiple	Multiple	Contraceptive use, reproductive autonomy, SRH knowledge, connectedness	N/A
Kelsey, et al., 2016 [69]	RCT	USA	English	Contraceptive use, sexual behavior	1809
Kennedy, et al., 2013 [70]	RCT	USA	English	Frequency of condom use, unprotected sex	136
Kerrigan, et al., 2008 [71]	Cross-sectional pre–post no control	Brazil	Unspecified	Community development, Condom use, unprotected sex	499
Kerrigan, et al., 2015 [72]	Systematic review & meta-analysis	Multiple	Multiple	STI and HIV infection rates, condom use	N/A
Kirby, et al., 2007 [73]	Systematic review	Multiple	Multiple	Initiation of sex, frequency of sex, number of sex partners, condom use, sexual risk taking, pregnancy rates, STD rates	N/A
Klein, et al., 2013 [74]	RCT	USA	English	Condom use, sexual communication self-efficacy	168
Klein, et al., 2017 [75]	RCT	USA	English Spanish	Condom use, STI rates, monogamous relationships, number of sexual partners, HIV & STI knowledge, partner communication, mental health	321
Kraft, et al., 2012 [76]	Cross-sectional post exposure	Zambia	Tonga Lozi	Number of sexual partners, condom use, HIV testing, perceived norms	1500
Krug et al., 2021a [77]	Quasi- experimental pre–post	Ethiopia	Not specified	Contraceptive use	1198
Krug, et al., 2021b [78]	Quasi- experimental pre–post	Nigeria	Not specified	Contraceptive use	18,950
Kudo, 2013 [79]	Quasi- experimental	Japan	Japanese	HIV/STD knowledge, SRH attitudes, risk perception, condom use, self-efficacy	280
Kuhlmann, et al., 2014 [80]	Cross-sectional dose-response	India	Not specified	Condom use, perceived fairness	1986
Kuo, et al., 2020 [81]	RCT	South Africa	Not specified	HIV risk behavior (condom use), depression	73
Lau, et al., 2014 [82]	RCT	China	Not specified	Consistent condom use	176
Lee, et al., 2009 [83]	RCT	USA	English Spanish	Condom use attitudes, Safer sex knowledge and skills, vulnerability to HIV, condom use	427
Lemani, et al., 2017 [84]	Cluster RCT	Malawi	Not specified	Contraceptive uptake	808
Lemieux, et al., 2008 [85]	Quasi- experimental nonequivalent control group pre–post	USA	English	HIV prevention knowledge, motivation, skills, and behavior, condom use	306
Lewin, et al., 2016 [86]	Prospective treatment- comparison	USA	English	Repeat pregnancy, contraceptive use	150
Lewin, et al., 2019 [87]	Prospective quasi- experimental	USA	English	Repeat pregnancy, contraceptive use	150
Li, et al., 2014 [88]	RCT	China	Not specified	Condom use	641
Lim, et al., 2019 [89]	Mixed-method quasi- experimental with control	Singapore	Not specified	Condom use	604-quant 10-qual
Logie, et al., 2014 [90]	Non-randomized cohort	Haiti	Kreyol	HIV knowledge, condom use, coping, depression, relationship control	200
Manlove et al., 2021a [91]	RCT	USA	English	Contraceptive use	1304
Manlove, et al., 2021b [92]	Cluster RTC	USA	English	Sexual experience, Condom use	626
Manlove, et al., 2022 [93]	Mixed-method individual randomized block no control	USA	English	Sexual risk behavior, condom use	51-quant 27-qual
Manlove, et al., 2020 [94]	RCT	USA	English	Contraceptive use	1304
Marcell, et al., 2013 [95]	Two-group quasi- Experimental pretest-posttest	USA	English	Knowledge about STDs, knowledge about health care, condom attitudes, sexual behavior (condom use), health use	197
Maticka-Tyndale, et al., 2007 [96]	Quasi- Experimental mixed-method pre–post	Kenya	Not specified	HIV knowledge, increased communication about HIV and sexuality, sexual activity, condom use	80
MEL Project. MEL of the Kenya Urban Reproductive Health Initiative (Tupange): Kenya, Endline Household Survey 2014, 2015 [97]	Longitudinal population level survey pre–post implementation	Kenya	N/A	Contraception use	15,337
Miller, et al., 2008 [98]	Quasi- Experimental separate sample pretest-posttest	Kenya	Not specified	HIV knowledge and attitudes, sexual behavior (condom use)	746
Moghaddam, et al., 2020 [99]	RCT	Iran	Not specified	Sexual satisfaction, marital satisfaction	70
Musa and Gegbe, 2013 [100]	Mixed-method Quasi- experimental	Sierra Leon	Not specified	Abstinence, monogamy, condom use, use of health services, livelihood activities	842-quant 44-qual
Norr, et al., 2012 [101]	Two group quasi- experimental pretest-posttest	Chile	Spanish	HIV attitudes, behavior (condom use) & knowledge, condom attitudes	555
Nanvubya, et al., 2022 [102]	RCT	Uganda	Luganda English	Contraceptive knowledge and use	1004
Pathfinder YUVAA Qualitative Assessment, Phase 3, Sustainability of Behavioral Change under YUVAA, 2022 [103]	Qualitative	India	Not specified	Contraceptive use, economic development	91
Peskin, et al., 2019 [104]	Group randomized waitlist controlled effectiveness trial	USA	English	Initiation of sexual behavior, contraceptive and condom use, number of sexual partners	1543
Philliber, 2021 [105]	RCT	USA	English	Condom use, safer sex knowledge, health care self- efficacy	1401
Potter, et al., 2016 [106]	Group RCT	USA	English	Initiation of sexual behavior, contraceptive use	3143
Prakash, et al., 2022 [107]	Quasi- experimental pre–post	Tanzania	Kiswahili	Contraceptive use	5043
Rensburg, 2007 [108]	Random sampling post exposure	South Africa	“Local Language”	Gender based violence attitudes, HIV/AIDS knowledge, condom use, abstinence, monogamy	304
Rohrbach, et al., 2015 [109]	Cluster RCT	USA	English	Sexual health knowledge, attitudes about relationship rights, partner communication, protection self-efficacy, sexual behavior (condom use)	1447
Rotz, et al., 2022 [110]	RCT	USA	English	Repeat pregnancy, father involvement, mother education and career aspiration, contraceptive use	594
Rotz, et al., 2018 [111]	Cluster RCT	USA	English	Sexual activity, condom use, SRH knowledge	1522
Sarafian, 2012 [112]	Pre–post no control	Bangladesh	Bengali	Condom use skills, knowledge & behavior, norms, self-efficacy	273
Scaling-up the Population, Health, and Environment Approach in the Lake Victoria Basin: A Review of the Results from Phases I and II of the Hope-LVB Project, 2018 [113]	Mixed-method observational post exposure	Kenya Uganda	Not specified	Successes and challenges to advancing reproductive health, contraceptive use, natural resource management	N/A
Sebany, et al., 2024a [114]	Cluster RCT Cross-sectional survey	Bangladesh	Bangla	Adoption and continuation of contraception	2308
Sebany, et al., 2024b [115]	Cluster RCT Cross-sectional survey	Tanzania	Kiswahili	Adoption and continuation of contraception	1129
Snyder, et al., 2014 [116]	Pre–post No control	South Africa	isiXhousa	HIV knowledge, attitudes and behavior (condom use)	109
Song, et al., 2014 [117]	Non-equivalent control group pre–post	South Korea	Not specified	Contraception knowledge & attitudes, sexual autonomy	156
Speizer, et al., 2018 [118]	Cluster randomized three-arm	South Africa	Not specified	Sexual risk, partner communication, conflict resolution, gender norms, alcohol use, condom use	299
Starosta, et al., 2016 [119]	RCT	USA	English	Condom use intentions, attitudes, and behaviors	422
Tebbets and Redwine, 2013 [120]	Mixed-method cross-sectional longitudinal no control	Ecuador Nicaragua	Spanish	Contraceptive use	597-quant 107-qual
Testing an Approach to Address Barriers to Family Planning for Postpartum Women Experiencing Depression and Anxiety, 2023 [121]	Qualitative post exposure	Ethiopia	Not specified	Mental health and wellbeing, barriers to contraception	26
Teti, et al., 2010 [122]	Mixed-method RCT	USA	English	Partner HIV disclosure, Condom use	184-quant 18-qual
Underwood, et al., 2012 [123]	Random selection Cross-sectional post exposure no control	Zambia	Not specified	Community capacity, contraceptive use, HIV testing	4816
Vazquez Guillamet, et al., 2024 [124]	Group randomized intervention trial	Cameroon	Pidgin English	HIV seroconversion, PrEP knowledge & interest, condom use	60
Visser, et al., 2020 [125]	Mixed-method post intervention no control	South Africa	English	Condom use confidence, knowledge & behavior,	1882-quant 243-qual
Visser, et al., 2007 [126]	Quasi- experimental stratified sampling pre–post	South Africa	English	Psychological wellbeing, personal control, school climate, HIV risk behavior (condom use)	2168
Wechsberg, et al., 2017 [127]	Two-group randomized trial	USA	English	Condom use	237
Wilson, et al., 2014 [128]	Pre–post No control	USA	English	Condom attitudes, behavior & self-efficacy, sexual risk behavior, community empowerment	80
Wong, et al., 2011 [129]	Pre–post No control	Hong Kong	Not specified	Sexual health knowledge, attitudes, and behavior	60
Yoshino, et al., 2023 [130]	Qualitative evidence synthesis	Multiple	Multiple	Health behaviors	N/A
Zellner, et al., 2015 [131]	Quasi- experimental	USA	English	Perceived stress, condom use, alcohol and drug use	192
Zief, et al., 2020 [132]	RCT	USA	English	Program exposure, contraception knowledge, contraception use, family functioning, child health and development	248

**Table 3:** Frequency of measurement of human flourishing constructs in VFP program evaluations that include at least 1 human flourishing metric.

Measured Construct	# of Articles	% of Articles
Self-efficacy	59	53.1%
Shared decision making	46	41.4%
Social support	44	39.6%
Mental health	23	20.7%
Agency	20	18.0%
Pleasure	18	16.2%
Empowerment	15	13.5%
Emotional intimacy	15	13.5%
Trust	14	12.6%
Social capital	11	9.9%
Autonomy	9	8.1%
Wellbeing	9	8.1%
Physical health	7	6.3%
Flourishing	5	4.5%
Meaning/purpose	5	4.5%
Happiness	2	1.8%
Valued	2	1.8%

## Data Availability

Data is available upon request from ABS.

## Supplementary Materials

The following supporting information can be downloaded at: https://www.mdpi.com/article/10.3390/ijerph23080998/s1, File S1: Preferred Reporting Items for Systematic reviews and Meta-Analyses extension for Scoping Reviews (PRISMA-ScR) Checklist.

## Abbreviations

The following abbreviations are used in this manuscript:

SRHR	Sexual and Reproductive Health and Rights
SRH	Sexual and Reproductive Health
VFP	Voluntary Family Planning
